# Calm Contact Technique Based on the Endocrinological Mechanism of Hypnosis—A Theoretical Proposal

**DOI:** 10.3390/brainsci14010083

**Published:** 2024-01-15

**Authors:** Katalin Varga, Zita S. Nagy

**Affiliations:** 1 Affective Psychology Department, ELTE Eötvös Loránd University, 1064 Budapest, Hungary; 2 MTA-ELTE Lendület Adaptation Research Group, Institute of Psychology, ELTE Eötvös Loránd University, 1053 Budapest, Hungary; 3 Mixed Profile Musculoskeletal Rehabilitation Department, National Institute for Medical Rehabilitation, 1121 Budapest, Hungary

**Keywords:** calm contact technique, stress reaction, oxytocin, social support, polyvagal theory

## Abstract

This paper proposes the “calm contact” technique: an imaginative scenario where someone is in gentle contact with a loved one where the essence of the experience is to enjoy safety and calmness in peaceful social contact. The theoretical background is outlined by combining the brain mechanisms of stress reactions and hypnosis. In addition to the ancient stress responses (flight or fight or freeze), there are oxytocin-based options at the human level: tend and befriend behavior and the state of calm and connection, which is not a stress reaction but a resting reaction. These social-based reactions could prevent the organism from the primitive freezing response. Some studies proved that “hypnosis” as a setting reduces cortisol levels and could raise oxytocin levels. The beneficial mechanisms of the “calm contact” technique are analyzed in relation to “social support” and the psychoaffective effects of central oxytocin. The subjective effects of the proposed technique are outlined based on reports of healthy volunteers. The “calm contact” technique could be an alternative or adjunct to the “safe place” technique, applying the recent findings of endocrinological brain mechanisms of hypnosis. Clinical implications and limitations are briefly summarized.

## 1. Introduction: Reactions to Stressors

A living organism, whatever its level of development, reacts as a whole to the stimuli and events it encounters. These reactions—at the human level—include behavioral, psychological (emotional and cognitive), and physiological processes.

We are highly sensitive to signs of danger in environmental stimuli. Subjective perception of danger activates one of the following three stress reactions, each of which has evolutionary roots:

1. The best-known stress reaction is the *flight or fight* reaction described in detail by Cannon [1] and Selye [2], one of the mobilization stress reactions. The danger that appears to be actively manageable activates the sympathetic nervous system, and as a result, the organism becomes active and alert. This physiological state enables organisms to have a fast reaction to danger: the fight or the flight.
2. The *tend and befriend* reaction described by Shelley E. Taylor [3] is another type of mobilization stress reaction. Organisms can react to danger with another kind of activation as well: finding partners who provide protection and/or taking care of offspring who require protection. While the *flight or fight* reaction is associated with increased production of adrenaline and cortisol at the hormonal level, the *tend and befriend* reaction is accompanied by an increased level of oxytocin [3].
3. The third type of stress reaction is the *freezing* reaction, which is one type of immobilization reaction. This stress reaction is evolutionarily more primitive than the two mentioned before [4]. The adaptive freezing reaction is activated only in life-threatening severe situations when the organism needs to survive at any cost, but there is no possibility for active coping. In this case, the organism responds with an increased function of the parasympathetic nervous system: the functioning of the body shuts down, and the organism becomes seemingly dead.

## 2. Safety and Calmness

In our everyday life, we are in almost continuous “stand by” mode. Usually, we are constantly monitoring our environment for potential dangers, and we are constantly looking for activities. That requires much energy, and it is also well known that this chronic state of stress could cause numerous severe health problems, e.g., cardiovascular disturbances, digestive disorders, and autoimmune diseases [4,5]. That is why the times when we feel safe are so precious. To find the psychological and physiological state of peace and quiet, one of the most commonly applied hypnotherapy techniques is the “safe place” technique [6,7,8]. This invites the patient to recall a safe place, imaginary or real, and enjoy the relaxed state.

In the case of mammals, especially humans, the reassurance, however, may come from the *social* partners. We instinctively search for the company of others in cases of high stress or danger. The natural situation in these cases is to have reassuring contact, e.g., holding a hand, touching the shoulder, or a hug. Already Harlow described the importance of “contact comfort”, especially in case of stressful events [9].

The complex cortical mechanism is responsible for managing the approach-avoidance response in a social context. When the situation is evaluated as “safe”, the “approach” response is activated, and a “calm” visceral state is associated [10]. A safe, warm, supportive social environment stimulates the state of *calm and connection* [11], which, unlike the stress reactions mentioned earlier, is a “resting reaction”. The significant calming effect of the safe social context is based on the neuroendocrine processes triggered by social support. There are more and more data regarding the beneficial effects of social support, which can be manifold, ranging from informational support through practical help to emotional support [10,12]. According to Porges, safe social connection activates the parasympathetic nervous system, which inhibits the activation of sympathetic nervous pathways [13]. This provides the neural basis of the calm visceral and psychological state. This “calm and connection” response, as well as the *tend and befriend* stress reaction, is modulated by oxytocin. Among the central effects of oxytocin (OT), we find the regulation of affiliation [14,15], reduction of fear and anxiety, and facilitating getting and providing social support throughout our whole life [16].

OT reactions are modulated by the social context [17,18]. Several aspects of social behavior are regulated by OT, like social cognition, memory of others, attention, and motivation processes [19]. OT is a natural mechanism to ease pain and manage stress. This works both in the mother and the newborn right after being born and delivered [20]. So, in an ideal case, we experience the possibility of reducing extreme stress in close contact with a loved one at the very beginning of our life. “Oxytocin, possibly acting via the autonomic nervous system, seems to play a central role in supportive relationships and psychologic safety, helping to protect and heal in the face of stress and adversity” [10]. It is hypostatized that being together with an empathic, supportive partner activates the oxytocin system [21]. The oxytocin system is activated by low-intensity physical contact (touch stroking) between adults and between animals and humans [22].

In addition, oxytocin is released in response to low-intensity stimulation of the skin, e.g., in response to touch, stroking, warm temperature, and so forth. Consequently, oxytocin is not only released during interaction between mothers and infants but also during positive interaction between adults or between humans and animals.

It is proven that apart from the “in vivo” human contacts, the mere *recall* or *imagined* version of a calm contact also raises the OT level [23].

Studies measure the peripheral OT change following traditional [24] and active-alert hypnosis [25]. In both forms, “hypnosis” as a setting reduced cortisol levels and could raise the OT levels of those whose baseline level was low (typically the low hypnotizable persons).

Several studies made the connection between OT and hypnosis [24,26,27]; the “*calm contact*” technique is a new association to activate by means of hypnotic suggestion the possibilities lying in gentle human contacts.

In this paper, we would like to propose a technique that is called “calm contact”. The purpose is that the participant/client enjoys the calmness-inducing, supposedly OT-activating effect of gentle, calm social contact.

We illustrate the effects of the technique by subjective reports given by participants in the exercise.

## 3. Materials and Method

### 3.1. Participants

During various university classes at Eötvös Lorand University (psychology, perinatal counselor, computer science), participation in the exercise was proposed on a voluntary basis in groups of 12–25 persons. All the participants were healthy students of higher education. No further data (gender, age) were registered to ensure anonymity. A blank sheet of paper was distributed to give a free anonymous report on the subjective effects of the exercise. We collected 53 reports. The quotations (see below) were selected and categorized by the authors.

### 3.2. Procedure

The “calm contact” technique exercise was delivered by the first author of this paper. There was no induction or deinduction. Participants were simply asked to take a comfortable position and close their eyes. The exercise lasted for 7 min.

### 3.3. Calm Contact Technique

*Please make yourself comfortable and recall a touch…, a hug…, a caress… that is especially dear and important to you, that gives you the most security… that makes you forget your worries… that feels good to snuggle into.*

*It may be connected to a real person or an imagined one… it could even be a combination of several of these experiences… recalling from one a mood… from another the feeling of touch… maybe a smell… from somewhere else, the experience of “everything is fine”…*

*It may be an experience from the past, even from so long ago that you have no conscious awareness of it… it may be a recent or even repeated, regular encounter… and it may be a memory of the future that has already arrived…*

*Sometimes you get it in the most unexpected situation… sometimes it’s not a person, but an animal, a cat or a dog…*

*The point is that the more you experience the comfort of the contact, the safety, and the security of being held… the more you can actually recharge yourself because your mind, your nervous system, your skin, and your muscles are storing the memory of the original experience…*

*This nice contact can increase your inner strength as if melting strength and power with calmness… it is good to know that you do have this possibility to strengthen your inner strength… meanwhile, you enjoy the nice calm contact…*

*With your breathing, you can further extend it… throughout your body… your soul…. so that it permeates everything… everywhere you need it, the experience of calmness and togetherness.*

*Just let it fill you… and in doing so, notice where you feel it the most… how it shows up… what represents that feeling… of peace, of calmness, of belonging…*

*Let this all come together as a symbol: it could be an image, a gesture, a body sensation, a word, or anything else. From now on, whenever you need the impact and support of that contact, you can call it forth by evoking that symbol.*

*…and now take a different breath and come back to the here and now…*

## 4. Results: Subjective Reports

### Usual and Unexpected Experiences

As expected, the exercise often induces a general feeling of calm, pleasure, refreshment, or distraction from unpleasant body sensations. It is also often the case that many modalities are condensed into the experience due to the altered state of consciousness.

“*I feel fresh; thanks for the experience*.”

“*I started smiling as I recalled the memories of those experiences, and it was a very good experience to be able to recall them like that*.”

“*It distracted me from my knee pain… it was relaxing, it was pleasant*.”

“*I had a bit of a headache at the beginning, but during the exercise, I felt it go away. I felt fresher by the end*.”

“*Minutes went by, but I am still very happy about this experience of being together*.”

“*I felt warmth, and it was as if, after a while, there was a complete envelope around me. The sensation was condensed into a glowing sphere and the melody and lyrics of a piece of music*.”

However, from personal accounts, it seems that unexpected experiences may also arise during the exercise. Sometimes, it’s difficult to maintain focus and stay in practice, and by focusing on the body, the experience can bring elements of the sympathetic stress response.

“*I am in a very confusing mental state lately, so trying to relax and focus on the mentioned instructions was hard for me. I was struggling to find what you were telling us for a bit, but after you said “cat”, I immediately found it in the feeling of the presence of my cat. Unfortunately, I could not maintain that contact with that sense of peace for a long time because I was so anxious, my heart was beating, and I was sweating like crazy*.”

“*When I had to spread the experience throughout the body, it was very pleasantly warm, but then somehow the experience became too much, and I started to discover more and more unpleasant bodily sensations. … So, at first, it was a very nice gentle shivering warmth, which then became a bit much and started to burn*.”

The positive feelings that arise during the exercise can also take many different, sometimes unexpected forms. For example, it may be accompanied by crying or by visual phenomena.

“*I was crying with the good feeling that I could have this, that I could have this feeling. It feels good to know that I have someone to rely on and that I am confident enough to not be ashamed of my feelings and to dare to own my tears*.”

“*The tears at the end of the exercise were an unexpected experience*.”

“*A flower came to my mind, with its petals closed and brushed against each other. These delicate petals will split when the time comes. The delicate petals are most closely related to the feeling I get when I’m hugged: security and tenderness*.”

The emergence of memories related to relevant others can also help recall the connected conflicts.

“*I never really felt safe with my mum*.”

“*This was difficult for me because I felt a strong lack within myself. During the pleasant hug, my little girl’s hug and smile came to my mind. I even smelled her scent, but it stopped me when safety came into play. Several times over the days, I have told myself and others that safety is exactly what is missing in my life and that it is not my daughter’s job to provide it. At this point, an image arrived of me curled up in a fetal position in someone’s lap, but there wasn’t any sense of body or even a sense of safety. For me, this was not a good experience (my husband moved away a few months ago)*.”

Some people actively resolve this conflict during the exercise using cognitive control.

“*…so I took the chance and went for the “it could be an animal” version, and I recalled the experience of petting my puppy. It was calming, but also it made me sad that it was easier to think of my dog than a human relationship*.”

Other times, the psyche helps to relieve the internal stress.

“*The human embrace was not reassuring; it made me anxious. That’s why colors came in as abstract images (green and purple)*.”

Sometimes, negative feelings might arise precisely because of these defense mechanisms:

“*Ideally, for me, the exercise is about the child-mother relationship, so I felt guilty that she should have shown up*.”

The experience is not always colored by positive emotions; for example, it can be combined with feelings of lack.

“*…When the two of us were standing in a room before the wedding, holding hands and crying because we were both thinking that we wished our mother was here, whose touch we missed the most*.”

“*I remembered that my father never hugged me*.”

In case of loss, it is the lack of a calming relationship that can dominate the experience, often with ambivalence.

“*Disharmony and contradictions were: pleasantness-frustration, closeness-distance, longing-repulsion, feels good-forbidden, it is gone-it stays forever*.”

“*The absence came as a tightness in my chest, a feeling of loneliness, and the thought that maybe no one would ever hold me like this. At times, I feel a feeling of falling and waking up, searching among people who will give me security. Doubt, am I able to feel safe?*”

“*The lack of a hug, which makes me sad because it is no longer there, and when it should have been, it wasn’t—although there are also positive experiences: the hug of a man, with whom it is good to rest, to relax, the happy hug and smile of a child*.”

Recalling the connection to people who have died can also trigger positive feelings:

“*My father, who has been dead for 22 years, clearly appeared. His hug was the safest hug I ever had in my life. It was a very strong hug, a long-lasting hug that I could relax into without words. There was a smell I loved about him when he smoked a pipe, the smell of pipe tobacco. It’s interesting that it came up, even though he hadn’t smoked for years at the end of his life. I loved that smell, the ritual of the way he filled the pipe. To add to that, his voice came to my ears, a very distinctive, strong, deep yet gentle voice*.”

“*The hug was the hug of my father, whom we lost five years ago. He was a big, strong man, and I imagined him and me standing in the hallway, him without a shirt (it must have been summer because he always wore a shirt). He hugged me tight but not hard. It was not an unpleasant experience, but sometimes I just feel how much I miss him. No one said anything while hugging. Basically, neither of us is the hugging type, but when I needed it, he was there and hugged me, and sometimes I hugged him too, just because*.”

In a therapeutic situation, these negative or ambivalent feelings and stressful experiences that arise during the exercise can provide a good starting point for further processing. For example, during the exercise, therapeutically important insights may emerge.

“*This is related to my mum. I didn’t receive her hug every time I needed it*.”

“*I realized how little time I spend connecting with them (mother and sister), although I really should do that for all three of us to get better*.”

The safety of a hug can come from many different sources: from a relationship with a mother/father, husband/wife, children, or the whole family, but it can also be linked to a close animal.

“*…and then in my mum’s lap, it smelt like it always did after a cigarette; I was little, but over the years as an adult, I’d crawl into her lap and experience the same old safe and secure feeling*.”

“*The hug was my husband’s hug, as he stood and hugged me while I snuggled in close, my head on his chest just where you could hear his heart beating very clearly. He is so calm. His two arms almost cover my back, and as he lowers his head, he touches the top of my head. So I can feel him on the top of my head and the feeling of “everything is going to be fine*.”

“*My husband’s body is the ultimate pleasure, an island of pleasure. As soon as you said what to do, in the blink of an eye, I was in his arms, and we were fused together without a “gap”, a feeling of all our cells coming together. I can smell every scent, and I feel his skin and every different bump on his skin at the end of my fingertip. I can almost feel his fingerprints. I don’t know how you can feel him so much. When I feel that, when we are all together like that, the world really does disappear. Not just in empty words, but really disappears. It sounds unbelievable, but there’s only this feeling and the millions of touching and heartfelt feelings. Just us. Just this. Total peace. Total calmness. Penetrating bliss. The two of us*.”

“*Laughing, the children run to me, M. and B.; I crouch down, and they run to me. They hug me and almost knock me over as they run to me. We laugh. I feel their silky hands on my skin, the touch of their soft skin. Their scent in my nose. Z. (dad) hugs me from behind, and we cuddle together. I feel warmth; it permeates my body*.”

“*It came to my mind when my dog snuggles up to me during our after-lunch siesta. He practically lies on me; he puts his neck against my neck. It’s a soft, empowering feeling to rest together. It is associated with the smell of nature and a great sense of freedom, release, and relaxation*.”

“*When I get to the lowest point in myself, I lie on the floor and hug my dog. I cry all my pain into his fur; there is the smell of home, the smell of my own care, the smell of unconditional and eternal love, the smell of protection. The countless memories of me lying on different floors and him always there holding me with his strong back, his paw on my shoulder, his big head tilted against mine, and I know we have each other*.”

The calm of a safe hug may sometimes come from the distant past or imagined future, and it may also be conflated with present experiences because of the altered state of consciousness.

“*Then I remembered a picture of my grandfather (he died when I was 11); I was sitting on his lap in the living room of their house, in the armchair. He’s hugging me with his big body; I’m quite small (about four years old). I captured that feeling in a Christmas tree ornament (snow globe). In my life, I have felt only with them that they really love me*.”

“*As the past was mentioned, that a hug could be a hug in which we were not yet self-aware, I thought of my state in the womb and then of the hug as my mum used to hold me when I was a baby*.”

“*And then I found even more peace in a vision of the future, a vision where we are husband and wife, our future is more secure than it is now, we can really connect, and I hug him, and I’m sure everything will be okay because he will stay with me and we will work everything out together*.”

“*The real peace that I feel when I fall asleep at night has come again: in our embrace, I imagine our not-yet-born-baby on my chest, the feel of his body against my skin, the warmth, his breathing, his heartbeat, and I get into a really relaxed state*.”

“*Snuggle together with my baby (2 months old), holding him on my shoulder + my own snuggle as a baby. Holding the baby on my shoulder, the heads come together, the two bodies “fit” together, mine and my baby’s (all four of my children) and my own body with my mother’s. Fitting together (merging), safe holding, the experience of holding in the whole body*.”

In the altered state of consciousness, the safe hugs associated with different people can combine into a collective, transpersonal, or even spiritual experience.

“*The long hugs, all of which were with people I loved, but the more I let myself in, the harder it was to distinguish whose hug I was feeling. Somehow, in the hugs I received from everyone, I found the one feeling they all had in common. It’s not the hug itself but the release after the hug as we look into each other’s eyes. That’s what’s really filling. When we feel that we have connected in a few moments and how much deep energy that has given us*.”

“*At first, I thought of many different hugs with different people, what it would feel like to hug someone smaller than me or someone bigger than me. Then I dwelled on the feeling of hugging and kissing a girl whose name I can’t remember. Then it became faceless; all the experiences filtered into one: we kissed, we hugged. It was warm, it was safe, it was glowing. It was past and future at the same time*.”

“*I feel myself very lovely and beloved. Also blessed by God*.”

“*I felt the touch of Jesus on my shoulder. I felt comforted; I knew I was safe and that the love that was flowing towards me would never end. There was no end to it, and I was grateful to have experienced it*.”

## 5. Discussion and Conclusions

The technique can be based on the recall of real-life contacts or a created/imagined one; it can be connected to a specific individual or a composition, including elements of various contacts.

The “calm contact” technique lends itself to combining it with the “safe/favorite place” technique. This way, we can utilize both the sense of physical and emotional safety. Social support is very beneficial both in acute and chronic stress, as it supports the restorative capacities of the body instead of the energy-consuming sympathetic response [10].

Emotional support is especially important when the stress is perceived as uncontrollable [28]. This means that social support provides far more than mere practical help. It actually transmits the message that “you are valuable and highly appreciated”, as the members of the community are paying attention and caring for you [29].

Studies of social support demonstrate that in some cases, when instrumental help is better, usually the family members are “called for,” while when someone requires joyful, positive encounters, peers and friends are needed [30,31]. In the “calm contact” technique, the patient (inner mind) can choose who is needed in the given situation.

We hypothesize that the “calm contact” technique can activate not only the experience of social support during stress but can also stimulate *calm and connection* reactions.

“Oxytocin appears to be of special relevance to physical and mental protective adaptations that involve high levels of sociality or social support and a sense of psychologic safety as well as emotional regulation and autonomic stability that are necessary for mental health and higher levels of cognitive functions”

[10] (p. 851).

Calm and connection reaction (OT-based stress regulation) has a specific psychophysiological pattern: low heart rate and blood pressure, gastrointestinal activation, reduced arousal, and increased sense of calmness. This state is related to a sense of well-being and relaxation and promotes positive social interaction [11], and it is especially important during childbirth and delivery [32].

According to Stephen W. Porges [4] and his Polyvagal Theory, the autonomic nervous system plays a prominent role in the regulation of reactions to the world. The model posits that three distinct neural pathways of the autonomic nervous system facilitate the production of different physiological states, which can be associated with different emotional states and behavioral responses. One of the central hypotheses of the Polyvagal Theory is that the regulation of social interactions is also related to the autonomic nervous regulation of the internal organs (especially the heart) because one of its neural pathways is a system with the cranial nerves (V, VII, IX, X, XI) that innervate the facial and masticatory muscles and the cephalic and middle ear muscles and thus play a crucial role in social interactions. The model, therefore, assumes that internal physiological states determine the possible forms of social behavior and thus considers a safe environment and the state of calm and connection as prerequisites for social connectedness.

OT facilitates a range of social skills: face recognition, peer recognition, emotion reading, empathy, trust, and generosity; it enhances communication adaptation by helping to match the partner’s ability to understand [10,33,34,35,36]. OT reduces social anxiety through the amygdala’s response to fearful faces [35]. The anti-anxiety effect is mediated through the parasympathetic branch of the autonomic nervous system: it reduces cortisol levels in the cardiovascular response to stress. This helps to promote a closer relationship with peers, which then supports the individual with peer forms of stress regulation. Peer support promotes the protective, restorative processes of the organism by blunting sympathetic tone [10]. The OT system activates the dopamine system, which provides the reward value of social cooperation and affiliation through its rewarding effects [34].

The calm contact technique, as it supposedly activates *calm and connection* reactions, may transform the current functioning of the nervous system and not only create a state of rest but also motivate social engagement.

The clinical relevance of the “calm contact” technique is that it can lead to adaptive affective-regulatory solutions by activating restorative capacities, social support, parasympathetic tone, and social engagement.

In clinical practice, “calm contact” technique has the specific advantage over existing imaginative internal safety techniques (e.g., relaxation, favorite/safe place) in activating secure modes of bonding. In a hypnotherapeutic situation, it is possible to follow the patient’s strengths and stumbling blocks in this area in process and even to use the flexibility of hypnotherapy to shape the repertoire of triggering situations and possible responses.

From this point of view, experiences that appear to be ‘unfavorable’ during demonstration can be turned into a therapeutic advantage. The “calm contact” technique seems to call up experiences of relational loss, insecurity, trauma, and, as several of the quoted reports of experiences have put it, can be spontaneously experienced during the technique. This can speed up their detection and correction in a therapeutic context.

The “hypnotic interaction”, according to Bányai’s social-psychobiological theory [37], serves as a model of our important relationships: the “calm contact” technique can open up a new level, where, in addition to the holding power of the actual bond with the living hypnotist, the imaginative way of correcting the difficulties of the relationship can be used. All this can be extended to real life, in hypno-vitro situations, to practice and polish the unfolding adaptive stress management situations [38].

Of particular interest, our research found both in traditional relaxational and active-alert hypnosis sessions that OT levels were elevated in hypnotists compared to the pre-hypnosis baseline—even though they were subjected to video recording in a foreign laboratory under restrained experimental conditions [24,25]. Of particular relevance to the hypnotist—and to the helping professions in general—is that OT can support the protection of the mental and somatic health of the professional.

## 6. Limitations and Future Possibilities

There is some evidence that the activation of the OT system is not always positive. Individuals who experienced trauma (especially in early life) might react adversely to exogenous OT, activating defensive responses instead of the *calm and connection* one [10] (Carter et al., 2020). Physical closeness and touch may generally be problematic for persons with a history of abuse. In these cases, special caution and (sense) of control need to be built in when applying the calm contact technique. The quotations of the reports connected to the technique illustrate that not only positive experiences are elicited. A well-trained instructor/therapist needs to handle the situation if needed.

Certain psychiatric and physiological conditions (e.g., high sympathetic stress levels, panic reactions, bodily dissociation), or being hypersensitive to internal bodily cues, can make it difficult to maintain focus, stay in practice and experience calm and safety during the calm contact procedure. In this case, a preliminary measurement of body awareness or the physiological symptoms of autonomic nervous system activity can help to support the exercise of the “calm contact” technique. Self-report questionnaires such as the Body Perception Questionnaire [39,40] can be used for this purpose.

The above outlined possible mechanisms regarding the OT response are theoretical suppositions that warrant empirical analysis.

There are many further questions to explore: How are hypnotic *interaction* and the Polyvagal Theory related? Can hypnosis be seen as an interaction in a resting state, thus refueling it as a “buffer” for emergencies? Can hypnosis be understood as being able to provide an adaptive solution to a stressful situation by modeling the stressful situation itself within a protected framework? Can we see hypnotic interaction as a reparative experience in place of possible adverse early experiences, in which the hypnotist provides a secure base for his subject?

So, the “calm contact” technique can enrich the repertoire of hypnotherapists and stimulate various research projects.

## Data Availability

The data presented in this study are available on request from the corresponding author. The data are not publicly available due to their qualitative nature.
